# Elephant-derived *Bacillus licheniformis* modulates immune cells shedding light on cancer resistance

**DOI:** 10.3389/fmicb.2026.1753410

**Published:** 2026-04-01

**Authors:** Xiaoyi Wang, Min Wei, Yang Yi, Jiaming An, Jingjing Wang, Rongjie Zhao, Zhan Su, Guozhong Ji, Xuemei Zhang, Xingyin Liu

**Affiliations:** 1 Core Facility Center, the First Affiliated Hospital of Nanjing Medical University, Nanjing, China; 2 Department of Pathogen-microbiology division, Key Laboratory of Pathogen of Jiangsu Province, Key Laboratory of Holistic Integrative Enterology, Nanjing Medical University, Nanjing, China; 3 Department of Gastroenterology, the Affiliated Jiangning Hospital of Nanjing Medical University, Nanjing, China; 4 Medical Center for Digestive Diseases, the Second Affiliated Hospital of Nanjing Medical University, Nanjing, China; 5 Department of Biochemistry, SUSTech Homeostatic Medicine Institute, School of Medicine, Southern University of Science and Technology, Shenzhen, China

**Keywords:** *Bacillus licheniformis*, colorectal cancer, elephant, gut microbiota, probiotic

## Abstract

**Introduction:**

Colorectal cancer (CRC) is currently a leading cause of cancer-related morbidity and mortality globally, underscoring the need for innovative therapeutic strategies. Probiotic treatment is increasingly appreciated as an innovative method for ameliorating inflammation and modulating the tumor microenvironment, especially in gastrointestinal diseases. Many bacterial species isolated from human and animal sources are proven effective in potential disease treatments. Elephants, renowned for their exceptional resistance to cancer, have traditionally been linked to their TP53 gene multiplicity. However, the potential contribution of their evolutionarily-refined gut microbiota to their remarkable cancer resistance remained largely unexplored.

**Methods:**

Here, we investigated this underexplored avenue by analyzing the elephant gut microbiome and isolating a probiotic bacterium. We utilized whole genome sequencing (WGS) to assess its genomic profile. The in vivo efficacy was evaluated in mouse models of gut inflammation and colorectal tumors. Underlying mechanisms were investigated using transcriptomic analysis, flow cytometry, and integrative metabolomics. Finally, in vitro experimental validations were conducted on mouse and human CRC cell lines using the bacterial culture supernatant.

**Results:**

We found that elephants possess a highly specialized gut microbiome finely tuned to metabolize complex polysaccharides. WGS of the isolated *Bacillus licheniformis* revealed its metabolic and functioning roles and confirmed the absence of virulence factors. We demonstrated that this elephant-derived strain effectively alleviated gut inflammation and suppressed the progression of colorectal tumors in mouse models. Transcriptomic analysis and flow cytometry revealed that *B. licheniformis* remodeled the immune microenvironment, specifically activating tumor-infiltrating T cell response and cell cytotoxicity. Integrative metabolomics identified several key metabolites as potential soluble mediators correlated with tumor regression. Furthermore, the supernatant of *B. licheniformis* culture significantly enhanced cytotoxicity and upregulated p53 expression in CRC cell lines *in vitro*.

**Discussion:**

Collectively, these findings unveil previously unrecognized therapeutic potentials inherent in elephant-derived probiotics, suggesting a mechanism of functional immune regulation for CRC prevention.

## 1 Introduction

Elephants exhibit exceptional cancer resistance despite their large body size and long lifespan, factors that typically increase cancer susceptibility (Abegglen et al., 2015). This phenomenon, known as Peto's Paradox, suggests that elephants possess unique biological mechanisms for cancer resistance (Vollrath, 2023; Nunney, 2022; Caulin and Maley, 2011). Previous studies have linked this resistance to their multiple copies of the TP53 tumor suppressor gene, which plays a critical role in DNA repair and apoptosis (Sulak et al., 2016; Casola, 2016). Emerging evidence highlights the gut microbiota as a key regulator of immune function and cancer prevention (Chen et al., 2025; Masaadeh et al., 2025). This complex microbial community shapes host immunity, metabolism, and inflammatory responses, which plays central roles in tumorigenesis. Genetic factors alone may not fully explain the extraordinary cancer resistance in elephants, raising the hypothesis that non-genetic factors, such as gut microbiota interactions contribute to their cancer protection.

Dysbiosis, characterized by microbial imbalance, has been strongly associated with colorectal cancer (CRC) development (Yang et al., 2021; Ternes et al., 2020). CRC ranks the third most prevalent cancer worldwide, accounting for approximately 10% of all cancer cases, and is the second leading cause of cancer-related deaths, with more than 1.9 million new cases reported in 2022 (Marcellinaro et al., 2023). Despite advances in surgical techniques, chemotherapy, and targeted therapies, the prognosis for advanced CRC remains poor due to therapy resistance and immune evasion within the tumor microenvironment (TME). These challenges underscore the urgent need for novel therapeutic strategies that can modulate the TME and overcome mechanisms of treatment resistance.

Probiotics have emerged as promising candidates for modulating gut health and cancer pathogenesis, offering a multifaceted approach to CRC prevention and treatment. Certain probiotic strains directly regulate the immune system, reducing colonic inflammation and enhancing antitumor immunity through various mechanisms, including production of bioactive metabolites (e.g., short-chain fatty acids), reinforcement of the gut barrier, and competitive exclusion of pathobionts. Specific strains, such as *Bifidobacterium* and *Lactobacillus*, can mitigate intestinal inflammation, inhibit tumor growth, and even improve responses to conventional therapies. However, the explorations of probiotics derived from exotic, disease-resistant animals remain in their infancy (Rosshart et al., 2017). Whether the unique microbiota evolved in elephant guts contributes to cancer resistance by enhancing immune function and inhibiting pro-tumorigenic pathways represents a novel and largely unexplored research direction.

In this study, we firstly investigated the characteristics of elephant gut microbiota and identified potential probiotic species unique to their microbiome. We isolated a strain of *Bacillus licheniformis* from elephant gut microbiota and examined its effects on colitis and tumorigenesis in multiple mouse models. Our findings demonstrate that this elephant-derived strain reduces tumor burden and suppresses colitis-associated carcinogenesis. These results establish a mechanistic connection between elephant microbiota and cancer resistance and highlight the therapeutic potential of host-adapted probiotics.

## 2 Materials and methods

### 2.1 Animals

Specific pathogen-free (SPF) male C57BL/6 (B6) mice (6–8-week old) were acquired from the Animal Core Facility of Nanjing Medical University and acclimatized for 7 days prior to experimentation. Animals were housed in a controlled barrier facility under SPF conditions, maintained at 23–26 °C and a 12-h light/dark cycle with free access to food and water. Mice were randomly assigned to different experimental groups based on initial body weight to ensure consistent baseline distribution. All animal experiments were approved by Institutional Animal Care and Use Committee (IACUC) of the Nanjing Medical University (Ethic No IACUC-2402037).

### 2.2 Anesthesia/Euthanasia methods

Mice were euthanized by cervical dislocation following deep anesthesia with tribromoethanol (avertin) at a standard dose of 250 mg/kg, in strict accordance with the AVMA guidelines for the Euthanasia of Animals and the Nanjing Medical University IACUC protocols. Death was confirmed by the absence of a pedal reflex and cessation of breathing. Target organs, including colorectal tumors, spleen, and intestines, were then aseptically dissected. Storage of tissue samples was performed by either snap-frozen in liquid nitrogen or fixed in 4% paraformaldehyde for subsequent analysis.

### 2.3 Bacterial strain and culture

A strain of *Bacillus licheniformis* was isolated from freshly collected elephant fecal samples obtained from Nanjing Hongshan Forest Zoo. Genomic DNA was extracted from the isolate, and the 16S rRNA gene was amplified via PCR using universal bacterial primers 27F and 1492R. The amplified product was purified and sequenced to confirm homology with *B. licheniformis* reference strains in the NCBI database. For routine culture, the isolate was streaked onto Luria-Bertani (LB) agar plates and incubated aerobically at 37 °C. Single colonies were inoculated into fresh LB broth and grown under agitation (200 rpm) at 37 °C for 16–24 h. Bacterial cells were harvested via centrifugation, washed twice and resuspended with sterile phosphate-buffered saline (PBS) solution for subsequent animal administration.

### 2.4 Acute colitis mice model induced by DSS

Male C57BL/6 mice (8 week-old) were used for acute colitis mice model and randomly assigned to different experimental groups. Acute colitis was induced by administering 2.5% (w/v) DSS (MP Biomedicals) dissolved in autoclaved drinking water for 7 consecutive days, followed by a 9-day recovery period with normal drinking water. For the *B. licheniformis* intervention group, mice were oral gavaged with 200 μL bacterial suspension (2 × 10^9^ CFU) every other day throughout the experimental timeline. This dosage was selected based on previous studies proven effective in murine models without adverse effects (Zhong et al., 2025). Control groups received equivalent volumes of sterile PBS in parallel. Body weights were monitored throughout the duration.

### 2.5 Subcutaneous tumor growth and treatment

MC-38 murine colorectal carcinoma cells were cultured in RPMI-1640 medium (Gibco) supplemented with 10% fetal bovine serum (FBS) and 1% penicillin-streptomycin at 37 °C under 5% CO_2_. For tumor implantation, 3–5 × 10^5^ cells were resuspended in 100 μL PBS and injected subcutaneously into the mouse right flank. Mice were randomly assigned to different experimental groups based on initial body weight to ensure consistent baseline distribution. Mice were orally gavaged with *B. licheniformis* (2 × 10^9^ CFU) every other day throughout the study, consistent with standard therapeutic dosing protocols for probiotic administration in mice (Zhong et al., 2025). Tumor dimensions were monitored throughout the duration using digital calipers in a blinded manner, and volumes were calculated as (length × width^2^)/2. Mice were sacrificed at the end of the experiment, with tumors and tissues collected, weighed, and processed for flow cytometry or RNA sequencing.

### 2.6 H&E staining and histological evaluation

Colons were fixed in paraformaldehyde, embedded in paraffin, sectioned, and stained with H&E. The histological pathology assessments for acute colitis and CRC were performed by blind reading of pathology slides according to published standards. Briefly, histological scoring criteria for colitis were based on severity of extent, damage, inflammation, and regeneration.

### 2.7 Flow cytometry assay

Single-cell suspensions were isolated from spleens and tumors for flow cytometry analysis according to previous protocol (Zhang et al., 2023). Briefly, spleen tissues were mechanically dissociated into single-cell suspensions and passed through a 70 μm cell strainer. The cell suspension was centrifuged and the pellet was resuspended in red blood cell lysis buffer (Huayunbio) for 3 min at room temperature, after which the reaction was neutralized by adding excess PBS containing 2% FBS and resuspended in RPMI 1640 supplemented with 10% FBS. The tumor tissues were gently fragmented using a MACS Octo Dissociator (Miltenyi Biotec, Bergisch Gladbach, Germany) and enzymatically digested in RPMI 1640 supplemented with 10% FBS, 1.5 mg/mL collagenase type VIII, and 100 U/mL DNase I (Sigma-Aldrich) at 37 °C for 30 min. Resulting suspensions were filtered through 70 μm strainers. The intermediate layer was collected after 80%/40% Percoll gradient centrifugation. Cells were placed into 96-well plates and fixed using Fixable Viability Stain 510 (BD Pharmingen) to exclude dead cells, followed by Fc receptor blockade with anti-CD16/CD32 (BioLegend, San Diego, CA, United States). Surface markers were stained with antibodies against CD45 (30-F11, BD Biosciences), CD3 (145-2C11, BioLegend), CD8 (53-6.7, eBioscience), CD4 (RM4-5, BioLegend), CD25 (PC61, BioLegend), CD11b (M1/70, BioLegend), and F4/80 (BM8, BioLegend). For intracellular and nuclear staining, cells were processed using the Foxp3/Transcription Factor Staining Buffer Set (eBioscience, San Diego, CA, United States) and stained with antibodies against Foxp3 (FJK-16s, eBioscience), IL-17a (TC11-18H10.1, BioLegend), and CD206 (C068C2, BioLegend). Cells for cytokine detection (IL-17a, IFN-γ) were stimulated with 100 ng/mL PMA, 1 μg/mL ionomycin, and 3 μg/mL brefeldin A (eBioscience) for 5 h (37 °C, 5% CO_2_). The gating strategies were defined as follows: Th17 cells (CD45+ CD3+ CD4+ IL-17a+), Tregs (CD45+ CD3+ CD4+ CD25+ Foxp3+), and M2 macrophages (CD45+ CD11b+ F4/80+ CD206+). Tumor-infiltrating cytotoxic T cells were defined specifically as CD45+ CD3+ CD8+ GZMB+. Single-stain controls were used to calculate the compensation matrix prior to analysis. Finally, samples were transferred into flow cytometry tubes, examined using a FACS Verse flow cytometer (BD Biosciences) and analyzed using FlowJo v10 (Tree Star).

### 2.8 *In vitro* cytotoxicity and gene expression assays

Mouse MC38 and human HCT116 colorectal cancer cell lines were cultured in DMEM medium supplemented with 10% FBS and 1% penicillin/streptomycin. LB medium and *B. licheniformis* culture supernatant were filter-sterilized (0.22 μm), freeze-dried and resuspended in DMEM medium. Resuspended culture medium is then added to the cancer cell culture medium at a concentration of 20% volume-by-volume (v/v) (labeled as 20% eBL). Cells were incubated for 24 h prior to analysis. Cell viability was assessed using CCK-8 assay, and apoptosis was detected via flow cytometry. Total RNA was extracted for RT-qPCR analysis of Trp53 expression as described above. Primer pairs 5'-ACATGACGGAGGTCGTGAGA and 5'-TTTCCTTCCACCCGGATAAG were used for Trp53 gene and primer pairs 5'-CATTGCTGACAGGATGCAGAAGG and 5'-TGCTGGAAGGTGGACAGTGAGG were used for ActB gene.

### 2.9 Whole genome sequencing (WGS) analysis

The genomic DNA of *B. licheniformis* was extracted using SDS and purification columns according to manufacturer's recommendations (Benagen Technology Co., Ltd., Wuhan, China). The quality and concentration of genomic DNA was measured using the Qubit 3.0 Fluorometer (Life Technologies, Carlsbad, CA, USA) and NanoDrop One Spectrophotometer (NanoDrop Technologies, Wilmington, DE, USA) before sequencing. We performed whole-genome sequencing of L168 using both the second-generation Illumina NovaSeq NGS platform and third-generation Nanopore sequencing technology. The sequencing data were assembled using Unicycler software, followed by alignment of the accurate short reads to the assembled contigs with Bowtie2. Contig corrections were made using Pilon software to reduce mismatches, insertions, and deletions. Genomic circular plots were generated using the R circlize package (Gu et al., 2020) to integrate and visualize the genome. Structural annotations (e.g., gene locations, repetitive elements), GC content distribution (calculated in sliding windows of 10 kb), and COG functional annotations (assigned via eggNOG-mapper v2.1.5) were formatted as input data layers. Specific metabolic pathways, including amino sugar and nucleotide sugar metabolism (Ko00520) and starch and sucrose metabolism (ko00500), were reconstructed to identify carbohydrate-active enzymes.

### 2.10 Metagenomic sequencing analysis

Metagenomic sequencing analysis was performed to characterize taxonomic profiles. Fecal samples were collected from a total of 15 Asian elephants. The cohort was stratified into two age groups: “Young” (ages < 18 years, *n* = 8) and “Old” (ages ≥18 years, *n* = 7) groups. Quality control and adapter trimming of raw FASTQ files were conducted using fastp with stringent parameters, including removal of low-quality reads, trimming of adapter sequences, and exclusion of short reads (Chen et al., 2018). All samples were sequenced in a single batch to minimize technical variation. Host DNA contamination was rigorously removed by aligning filtered reads to Asian elephant reference genome (*Elephas maximus* EmaxH1_LGv1.0) using Bowtie2 (v2.5.1) with unmapped reads retained for downstream microbial profiling (Langmead and Salzberg, 2012). Taxonomic classification was performed using Kraken2 (v2.1.2) with the standard reference database (RefSeq archaea, bacteria, viral, and plasmid genomes) with default parameters (Wood et al., 2019). Taxonomic abundances were estimated from Kraken2 reports using Bracken (v2.8) (Lu et al., 2017). Statistical analyses of microbial diversity (alpha: Shannon diversity) and beta diversity (Bray-Curtis) were conducted in R (v4.3.1) using phyloseq (McMurdie and Holmes, 2013) and vegan packages. Differential taxa between old and young groups were plotted using pheatmap package.

### 2.11 Transcriptomics (RNA-seq) analysis

Total RNA was extracted from bulk tumor tissues (*n* = 4 biological replicates per group) using Trizol reagent (Life science). RNA concentration and integrity was confirmed by Agilent 2100 Bioanalyzer. RNA-Seq analysis was performed with an Illumina Novaseq6000 (PE150) by Novogene Company (Tianjin, China). To ensure accurate RNA sequencing data were processed to identify transcriptomic changes and associated biological pathways. Raw paired-end reads were quality-filtered and adapter-trimmed using fastp (v0.23.4) with stringent thresholds (Phred score ≥ 20, read length ≥ 50 bp). High-quality reads were aligned to the Mus musculus reference genome (GRCm39/mm39) using HISAT2 (v2.2.1) (Kim et al., 2019) and quantified at the gene level with featureCounts (v2.0.3) using GENCODE annotations (vM33) (Liao et al., 2014). Differential expression analysis was performed in DESeq2 (v1.40.2) with default parameters, identifying genes with significant expression changes (adjusted *p* < 0.05, |log2 fold change| > 1) (Love et al., 2014). Results were visualized as volcano plots using ggplot2 (v3.4.4), highlighting differentially expressed genes (DEGs) and their statistical significance. Top DEGs were also plotted as a heatmap using the pheatmap (v1.0.12) package. Functional enrichment analysis of DEGs was conducted with ClusterProfiler (v4.8.3), testing for overrepresentation of Gene Ontology (GO) biological processes and Kyoto Encyclopedia of Genes and Genomes (KEGG) pathways (FDR < 0.05) (Yu et al., 2012). Gene Set Enrichment Analysis (GSEA) was additionally performed to identify non-thresholded pathway-level trends, ranking all genes by log2 fold change and testing against the Molecular Signatures Database (MSigDB) hallmark gene sets (v7.5.1) using a permutation-based FDR correction (Subramanian et al., 2005).

### 2.12 LC-MS/MS metabolomics analysis

To identify bioactive metabolites secreted by the probiotic, *B. licheniformis* was cultured in LB broth for 12 h (37 °C, 200 rpm). The culture was centrifuged at 12,000 rpm for 10 min at 4 °C and the supernatant was filtered through a 0.22 μm membrane. Sterile, uninoculated LB broth processed identically served as the control with four biological replicates for each group. For metabolite extraction, 100 μL of supernatant was mixed with 400 μL of ice-cold extraction solvent (methanol: acetonitrile: water, 2:2:1, v/v/v) containing isotopically labeled internal standards. The mixture was vortexed for 30 s, sonicated for 10 min at 4 °C, and incubated at −20 °C for 30 min to precipitate proteins. After centrifugation (12,000 rpm, 15 min, 4 °C), the supernatant was collected and dried in a vacuum concentrator. Samples were reconstituted in 100 μL of starting mobile phase (99% water + 1% acetonitrile + 0.1% formic acid, v/v/v) prior to analysis.

Chromatographic separation was performed using a Vanquish UHPLC system (Thermo Fisher Scientific, Waltham, MA, United States) equipped with an ACQUITY UPLC HSS T3 column. The mobile phases consisted of 0.1% formic acid in water (A) and 0.1% formic acid in acetonitrile (B). Mass spectrometry was performed on a Q Exactive HF-X Hybrid Quadrupole-Orbitrap mass spectrometer (Thermo Fisher Scientific) operating in both positive and negative electrospray ionization (ESI) modes. Data processing and metabolite identification raw data files were processed using Compound Discoverer 3.1 for peak alignment, retention time correction, and peak area extraction. After acquiring feature table and compound names, downstream analysis was performed by R (v4.4.1).

### 2.13 Statistical analysis

All statistical analyses were conducted by R (v4.4.1) and GraphPad Prism 9.0 software. All data are represented as the mean ± SEM. Sample sizes (n) for each experiment are explicitly stated in the figure legends. Bacterial taxonomic comparisons were conducted using Wilcoxon rank sum test between two groups. For comparisons among three or more groups (e.g., DSS colitis parameters), One-way ANOVA followed by Tukey's multiple comparison *post-hoc* test was employed. Tumor growth curves were analyzed using a Two-way Repeated Measures ANOVA to account for time-dependent interactions. *P-values* were corrected with the Benjamini-Hochberg method when appropriate. ^*^P < 0.05, ^**^P < 0.01, ^***^P < 0.001, ^****^P < 0.0001 and n.s. indicates not significant (*P* > 0.05).

## 3 Results

### 3.1 Elephant gut microbiota harbors a rich community of cellulose-decomposing bacteria

**Figure 1:**
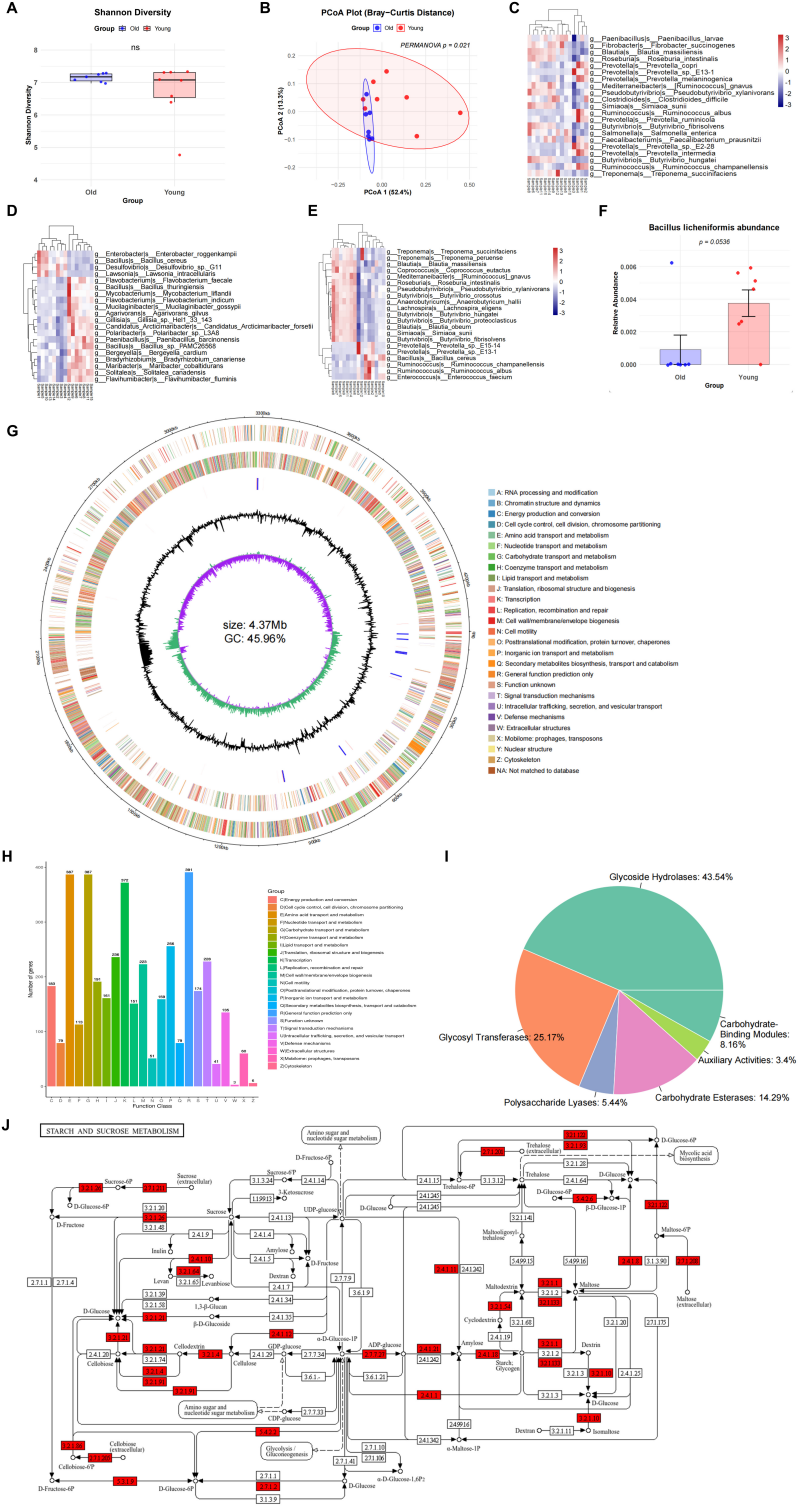
Metagenomic and functional analysis of elephant gut microbiota. **(A)** Alpha diversity (Shannon index) in young (*n* = 8) and old (*n* = 7) elephant groups. Data are presented as mean ± SEM. Statistical significance determined by Wilcoxon rank-sum test. **(B)** Beta diversity using PCoA analysis in young and old elephant groups. **(C)** Top abundance bacterial species identified in elephant fecal microbiome. **(D)** Top differential bacterial species (ranked by *P-values*) in abundance identified between young and old elephant fecal microbiome. **(E)** Top differential bacterial species (ranked by relative abundances) in abundance identified between young and old elephant fecal microbiome. **(F)** Relative abundance of *B. licheniformis* in young and old elephant groups (Wilcoxon rank-sum test). **(G)** Whole genome sequencing of *B. licheniformis* genome. **(H)** Biological processes and functional annotations for *B. licheniformis* genome. **(I)** *B. licheniformis* genes identified in carbohydrate-active enzyme (CAZy) database. **(J)** *B. licheniformis* functional enzymes identified starch and sucrose metabolism (ko00500).

Elephants are extremely cancer-resistant species due to genetic and non-genetic factors. Gut microbiota may contribute to their cancer resistance. Elephants are herbivores that consume a variety of plants, including grasses, leaves, fruits, and tree barks high in fiber and low in protein (Dierenfeld et al., 2020). We infer that the gut of elephants likely harbors a rich community of cellulose-decomposing bacteria fine-tuned for digesting the high-fiber content of their diet. These bacteria can break down cellulose and other plant polysaccharides into nutrients like short-chain fatty acids that elephants can absorb. Some of these bacteria may be persistent gut symbionts that remain in the elephant's gut throughout different ages. To explore persistent gut symbiotic bacteria across different ages in elephants and whether certain species have the potential function to inhibit inflammation and tumor growth, we first harvested fecal samples from elephants and performed metagenomic sequencing analysis. Taxonomic analysis showed no differences in alpha diversity between young and old elephant groups, but beta diversity revealed distinct compositional structures (Figures 1A, B). Consistent with the herbivorous diet of the host, the elephant gut harbors a rich community of persistent cellulose-decomposing bacteria, including *Prevotella, Faecalibacterium* and *Ruminococcus* (Figure 1C). Furthermore, we compared differential abundance between young and old groups and observed that some species, such as *Bacillus, Prevotella*, and *Ruminococcus* species showed decreased abundance along with aging (Figures 1D, E). These bacteria are known for association with high-fiber, non-Western diets, production of short-chain fatty acids and anti-inflammatory roles. These bacteria are prevalent members of the gut microbiota that plays crucial roles in the digestive systems, especially in breaking down complex plant fibers and plant carbohydrates (Prasoodanan et al., 2021; Yohannes et al., 2020).

We further identified a specific bacterial species within the *Bacillus* genus, *B. licheniformis*, which negatively correlates with age (Figure 1F) and managed to isolate a *B. licheniformis* strain from elephant feces. *B. licheniformis* can regulate gut microbiota balance and promote the growth of several beneficial bacteria, such as *Roseburia, Bifidobacterium*, and *Coprococcus* (Feng et al., 2022). It inhibits harmful bacteria by producing antibacterial active substances and through its unique biological oxygen-depleting mechanism, thus maintaining the microecological balance of the gut microbiota. We then performed whole genome sequencing of this isolated strain to further explore its physiological and biochemical functions. Consistent with the host's high-fiber herbivorous diet, the genome revealed a robust “wood-degrading” enzymatic module. *B. licheniformis* possesses robust structures of protease, lipase, and amylase, indicating that elephants can effectively utilize them to promote the degradation of nutrients in the intestine and enhance the utilization of cellulose (Figures 1G, H). Importantly, it possesses essential enzymes that utilize diverse plant-derived carbohydrates, such as cellulose and cellobiose along with arabinose and xylose (Supplementary Figure S1). Specifically, we identified key genes encoding endoglucanase (EC 3.2.1.4) and cellulose 1,4-beta-cellobiosidase (EC 3.2.1.91), which function synergistically to depolymerize complex cellulose fibers into cellobiose (Figure 1J). In addition to cellulolytic capabilities, the strain exhibits broad polysaccharidase activity, possessing alpha-amylase (EC 3.2.1.1) and neopullulanase (EC 3.2.1.133) for starch degradation, as well as levansucrase (EC 2.4.1.10), which converts sucrose into levan, a fructan known for its prebiotic and immunomodulatory properties (Figure 1I) (Young et al., 2021). In summary, the findings implicated that gut microbiota of elephants might specialize in the digestion of polysaccharides in their high-fiber diet. Moreover, these bacteria including *B. licheniformis* produce anti-inflammatory short-chain fatty acids, which contribute not only to their digestive health but also to their overall wellbeing, highlighting the importance of gut microbiota in maintaining elephant health (Feng et al., 2022).

### 3.2 *B. licheniformis* alleviates gut inflammation in mouse models

**Figure 2:**
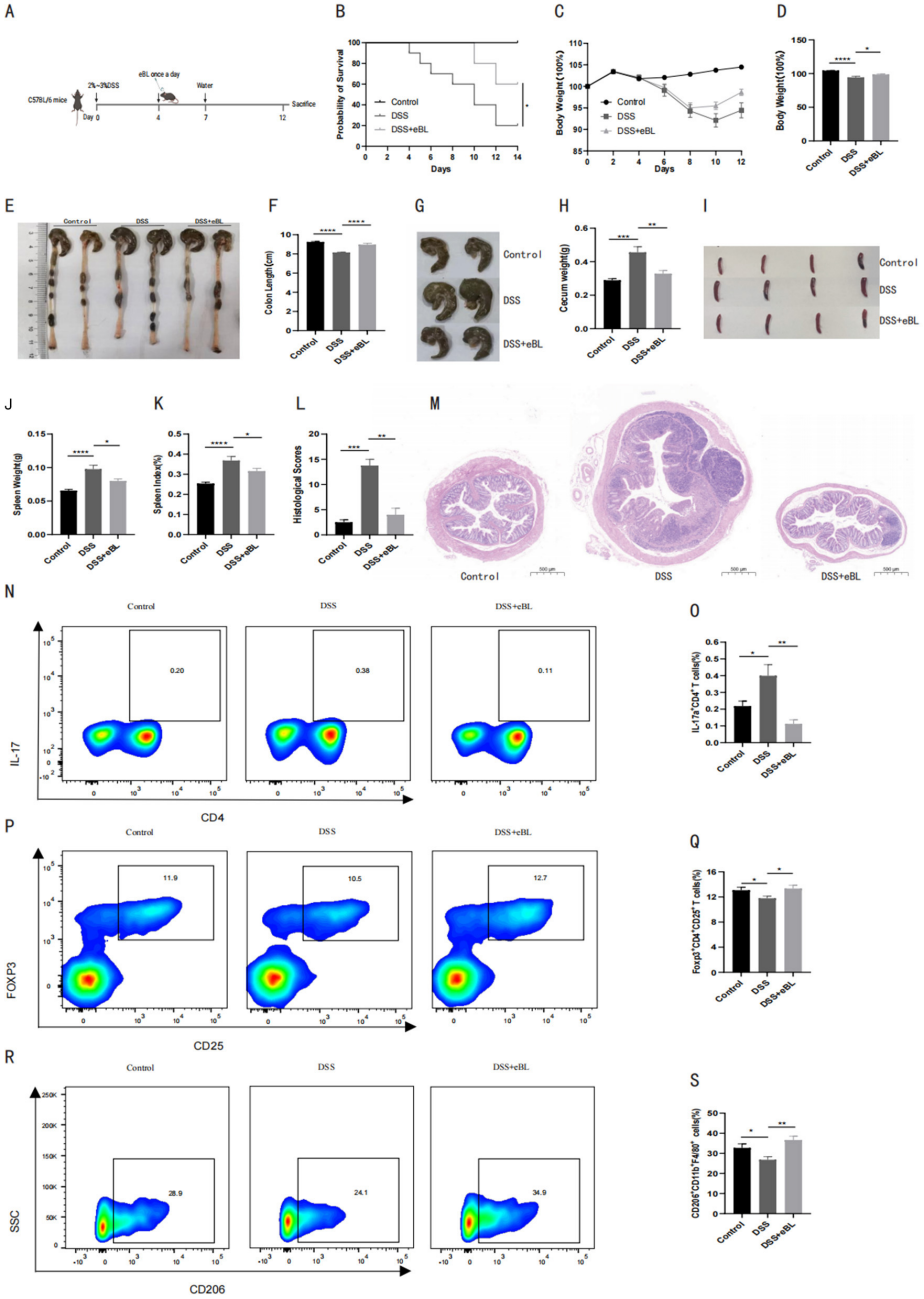
*B. licheniformis* ameliorated gut inflammation of acute colitis in mice (*n* = 20 biologically independent mice per group). **(A)** Representation of acute colitis mouse model. **(B)** Survival probability of mice in control, DSS and treatment groups. **(C)** Changes in body weight of mice in control, DSS and treatment groups (Two-way Repeated Measures ANOVA with Tukey's multiple comparisons test). **(D)** Changes in body weight of mice in control, DSS and treatment groups at the end of experiment. **(E)** Representative images of colon in control, DSS and treatment groups. **(F)** Length of colons in control, DSS and treatment groups. **(G)** Representative images of cecum in control, DSS and treatment groups. **(H)** Weights of cecum in control, DSS and treatment groups. **(I)** Representative images of spleens in control, DSS and treatment groups. **(J)** Spleen weights in control, DSS and treatment groups. **(K)** Spleen index in control, DSS and treatment groups. **(L)** Histology scores of colon H&E staining in control, DSS and treatment groups. **(M)** Representative images of colon H&E staining in control, DSS and treatment groups. **(N)** Flow cytometry of IL-17+ T cells in control, DSS and treatment groups. **(O)** IL-17+ T cell percentages in control, DSS and treatment groups. **(P)** Flow cytometry of Treg cells in control, DSS and treatment groups. **(Q)** Treg percentages in control, DSS and treatment groups. **(R)** Flow cytometry of M2 macrophages in control, DSS and treatment groups. **(S)** M2 macrophage percentages in control, DSS and treatment groups. **Data are presented as mean ± SEM. Endpoint comparisons (d-s) were analyzed using One-way ANOVA followed by Tukey's *post-hoc* test. *P < 0.05, **P < 0.01, ***P < 0.001, ****P < 0.0001.

*B. licheniformis* is a well-established probiotic species often utilized for its anti-inflammatory properties, particularly in the context of gastrointestinal disorders such as diarrhea and inflammatory bowel diseases (Ramirez-Olea et al., 2022). We first investigated the therapeutic potential of the elephant-derived *B. licheniformis* isolate in a dextran sulfate sodium (DSS)-induced colitis mouse model (Figure 2A). We monitored survival and body weight changes throughout the experiment. DSS administration significantly reduced survival and body weights compared to the control group, indicating the onset of severe colitis. Oral administration of *B. licheniformis* resulted in a marked recovery in both parameters (Figures 2B–D).

Furthermore, the treatment ameliorated macroscopic disease indices, including colon length shortening and spleen enlargement, and spleen index were all significantly improved in the *B. licheniformis*-treated groups compared to the DSS-only group (Figures 2E–K). Histological assessment (blinded scoring) confirmed that *B. licheniformis* preserved colonic architecture and reduced inflammatory infiltration (Figures 2L, M). These findings indicate that *B. licheniformis* not only protects against the loss of body weight but also reduces the extent of colon damage, further supporting its promising therapeutic potential in mitigating gut inflammation.

To elucidate the immunological mechanism, we analyzed lymphocyte populations via flow cytometry. In DSS-treated mice, the Th17/Treg balance was disrupted, favoring pro-inflammatory Th17 cells. *B. licheniformis* treatment significantly reversed this trend, suppressing Th17 cells while expanding the population of Foxp3+ Regulatory T cells (Tregs) and anti-inflammatory M2 macrophages (Figures 2M–S), thereby restoring immune homeostasis in the inflamed gut.

We then proceeded to analyze pro-inflammatory and anti-inflammatory immune cell populations to investigate underlying immunological mechanisms using flow cytometry. In the DSS-treated groups, there was a significant increase in the number of IL17a+ CD4+ T (Th17) cells, a key player in driving inflammation. However, oral administration of *B. licheniformis* significantly reduced the levels of Th17 cells, indicating a suppression of pro-inflammatory responses (Figures 2K, L). In parallel, we observed a decrease in regulatory T cells (Tregs) and M2 macrophages, which are critical for maintaining immune tolerance and reducing inflammation, in the DSS-treated group. Notably, *B. licheniformis* oral treatment reversed this trend, increasing the levels of Tregs and M2 macrophages and restoring balance in Th17/Treg cells (Figures 2M–S). Collectively, these findings demonstrate that *B. licheniformis* alleviates gut inflammation in the DSS-induced colitis mouse model, reducing pro-inflammatory Th17 cells and promoting anti-inflammatory Tregs and M2 macrophages.

### 3.3 *B. licheniformis* alleviates subcutaneous colorectal tumor growth by enhancing various types of immune cells in mice

**Figure 3:**
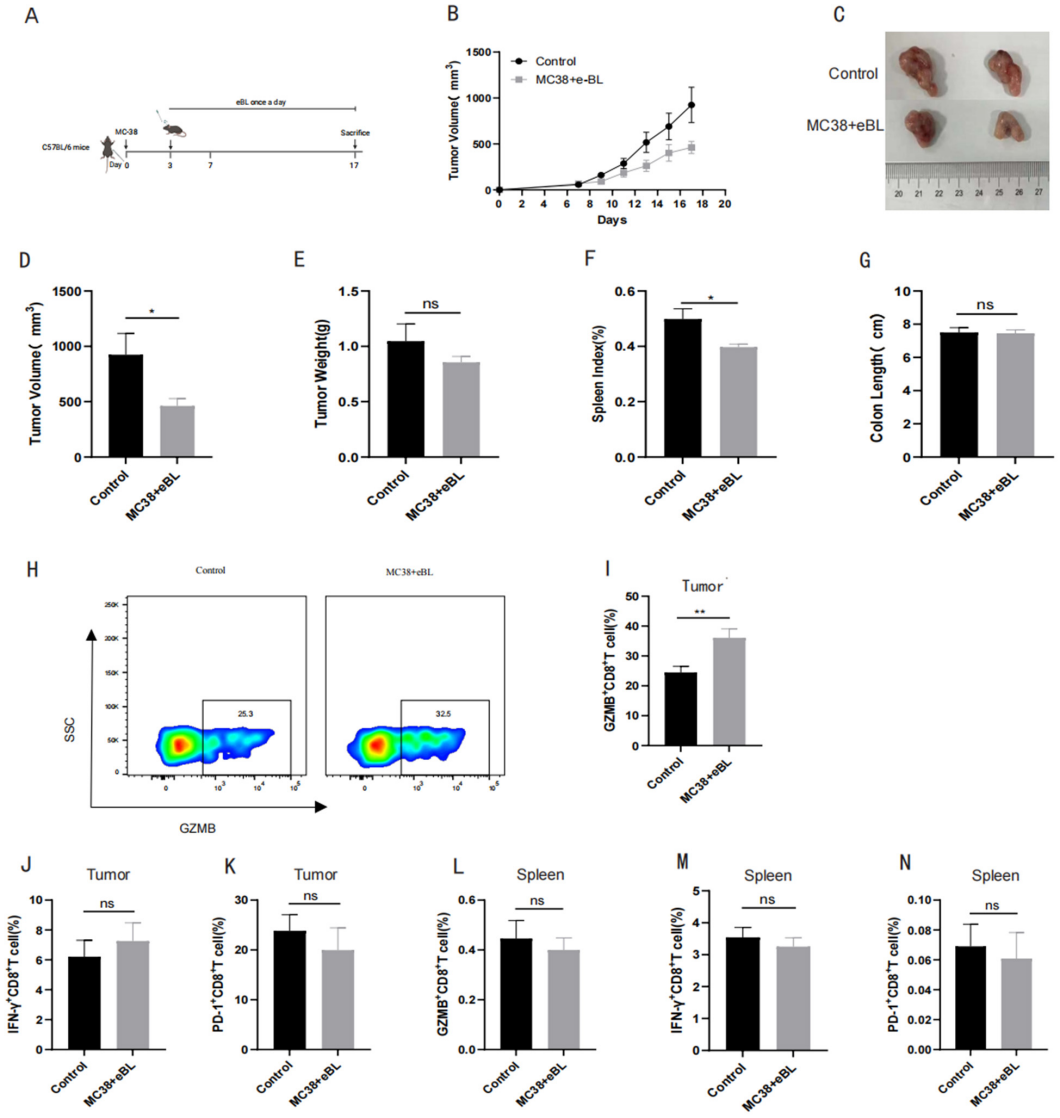
*B. licheniformis* administration enhanced CD8+ T cell cytotoxicity against subcutaneous tumor growth (*n* = 20 biologically independent mice per group). **(A)** Representation of MC38 subcutaneous tumor mouse model. **(B)** Growth of tumor volumes in control and treatment groups. **(C)** Representative images of tumors in control and treatment groups. **(D)** Tumor volumes in control and treatment groups. **(E)** Tumor weights in control and treatment groups. **(F)** Spleen index in control and treatment groups. **(G)** Colon length in control and treatment groups. **(H)** Flow cytometry of GZMB+ CD8+ T cells in tumor tissues in control and treatment groups. **(I)** Percentages of GZMB+ CD8+ T cells in tumor tissues in control and treatment groups. **(J)** Percentages of IFN-γ+ CD8+ T cells in tumor tissues in control and treatment groups. **(K)** Percentages of PD-1+ CD8+ T cells in tumor tissues in control and treatment groups. **(L)** Percentages of GZMB+ CD8+ T cells in spleen in control and treatment groups. **(M)** Percentages of IFN-γ+ CD8+ T cells in spleen in control and treatment groups. **(N)** Percentages of PD-1+ CD8+ T cells in spleen in control and treatment groups. **Data are presented as mean ± SEM. Endpoint comparisons (D-N) were analyzed using unpaired Student's *t*-test. *P < 0.05, *P* < 0.01. ns indicates not significant (*P* > 0.05).

To further explore whether *B. licheniformis* exerts therapeutic effects in CRC, we established subcutaneous tumor xenograft models by injecting MC-38 colorectal cancer cells into mice. The animals were then orally gavaged with either *B. licheniformis* or PBS solution as a control (Figure 3A). Changes in tumor sizes were monitored over the course of the experiment and the mice were sacrificed at the endpoint to analyze tumor progression. As shown in both tumor volume and weight measurements, oral administration of *B. licheniformis* significantly inhibited tumor growth compared to the PBS-treated control group (Figures 3B–E). These results suggest that *B. licheniformis* has a suppressive effect on the growth of subcutaneous colorectal tumors. In addition to assessing tumor size, we also examined the impact of *B. licheniformis* on immune cells and functions in the spleen and colon. We observed that the spleen index was significantly improved in the *B. licheniformis*-treated group (Figures 3F, G), indicating a potential enhancement of immune system activity. However, no significant differences in colon pathology or immune cell infiltration were observed between the groups.

Given the critical role of immune cells in anti-tumor immunity, particularly cytotoxic CD8+ T cells, we then focused on examining the effects of *B. licheniformis* on various subtypes of CD8+ T cells within the tumor microenvironment. One of the most striking findings was the significant increase in the percentage of GZMB+ CD8+ T cells in the tumor region following *B. licheniformis* administration (Figures 3H, I). Granzyme B (GZMB) is a key cytotoxic marker for CD8+ T cells, indicating that *B. licheniformis* enhances the recruitment or activation of tumor-specific CD8+ T cells capable of inducing tumor cell death. This suggests that *B. licheniformis* may stimulate an effective anti-tumor immune response by promoting cytotoxic T cells in the tumor microenvironment. While we observed significant changes in the GZMB+ CD8+ T cell population, other markers associated with CD8+ T cell activation, such as IFN-γ+ and PD-1+ CD8+ T cells, were not significantly altered in the tumor region (Figures 3J, K). This indicates that while *B. licheniformis* may enhance cytotoxic activity (as evidenced by GZMB expression), it may not significantly influence the overall activation or exhaustion states of CD8+ T cells, as indicated by IFN-γ and PD-1 levels.

We also examined the impact of *B. licheniformis* on CD8+ T cell subtypes in the spleen, a key secondary lymphoid organ where immune cells are primed and activated. However, in the spleen, no significant differences were observed in the percentage of GZMB+, IFN-γ+, or PD-1+ CD8+ T cells between the *B. licheniformis* and PBS-treated groups (Figures 3L–N). This suggests that *B. licheniformis* may specifically influence the tumor microenvironment rather than inducing systemic changes in CD8+ T cell populations, highlighting a localized effect of the probiotic on anti-tumor immunity. Collectively, these results suggest that *B. licheniformis* inhibits subcutaneous colorectal tumor growth through the enhancement of tumor-infiltrated GZMB+ CD8+ T cells, which are essential for cytotoxic anti-tumor activity. The lack of significant changes in other CD8+ T cell markers, such as IFN-γ and PD-1, suggests that *B. licheniformis* may promote a more targeted or early cytotoxic immune response in the tumor microenvironment.

### 3.4 *B. licheniformis* supernatant enhances cytotoxicity and p53 expression in mouse CRC cells

**Figure 4:**
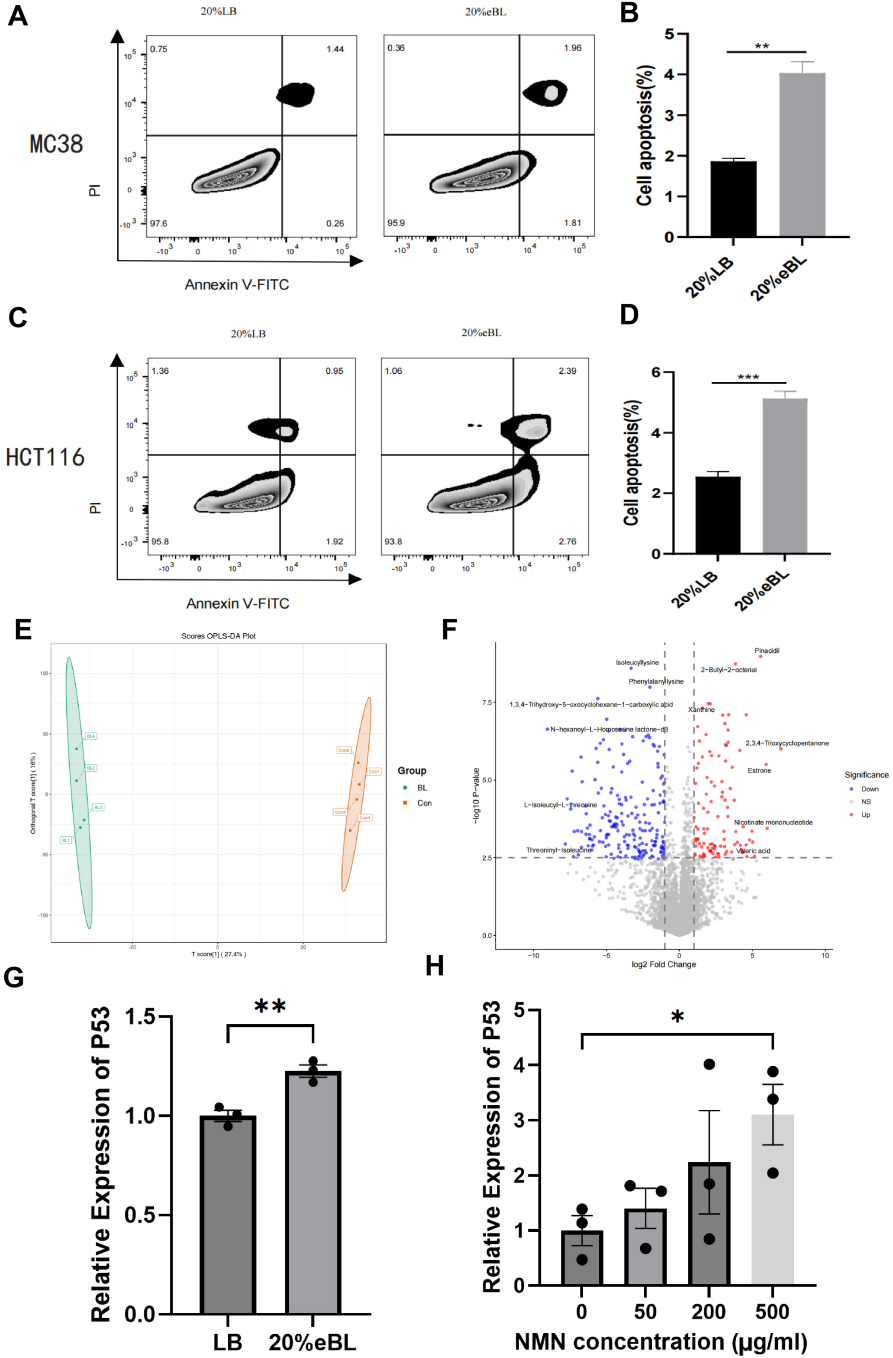
*B. licheniformis*-derived metabolites enhanced tumor apoptosis and TP53 expression in tumor cell lines (*n* = 3 biological replicates). **(A, B)** *B. licheniformis* culture supernatant induced cell apoptosis in MC38 cells. **(C, D)** *B. licheniformis* culture supernatant induced cell apoptosis in HCT116 cells. **(E)** PCA plots shows that *B. licheniformis* produces unique metabolites compared to blank medium. **(F)** Volcano plots showed specific metabolites are accumulated compared to blank medium. **(G)** *B. licheniformis* culture supernatant (20% v/v) induced P53 gene expression in MC38 cells. **(H)** NMN induced P53 gene expression in MC38 cells. *P < 0.05, **P < 0.01, ***P < 0.001.

Having established the anti-tumor effects of *in vivo* administration of *B. licheniformis*, we sought to investigate its direct impact on colorectal cancer cells *in vitro*. We treated both mouse and human CRC cell lines with the filter-sterilized supernatant of *B. licheniformis* culture and observed that *B. licheniformis* treatment significantly induced cell apoptosis of MC38 and HCT116 cells (Figures 4A–D). Next, we collected the supernatant and performed untargeted metabolomic analysis to identify specific *B. licheniformis*-derived metabolites (Figure 4E). We found that certain SCFAs especially valeric acids and nicotinate mononucleotide (NMN) are also significantly accumulated (Figure 4F). This molecule has shown potential in treating and alleviating many human diseases and disorders. To support this finding, metabolic pathway analysis was also performed to confirm that *B. licheniformis* possesses essential genes for NMN and NAD+ biosynthesis pathways (Supplementary Figure S2).

Given the importance of p53 in elephant cancer resistance and the differential effect we observed, we next examined the gene expression level of p53. RT-qPCR analysis showed MC38 cells treated with *B. licheniformis* supernatant showed remarkably higher P53 expression compared to the untreated counterpart (Figure 4G). Consistently, NMN treatments also induced P53 expression in MC38 cells (Figure 4H). These results indicate that *B. licheniformis* produces soluble factors that can directly induce cytotoxicity in CRC cells and upregulate the tumor suppressor p53, providing a potential direct mechanism for its anti-cancer effects alongside its immunomodulatory properties.

### 3.5 RNA-seq analysis reveals activation of immune pathways

**Figure 5:**
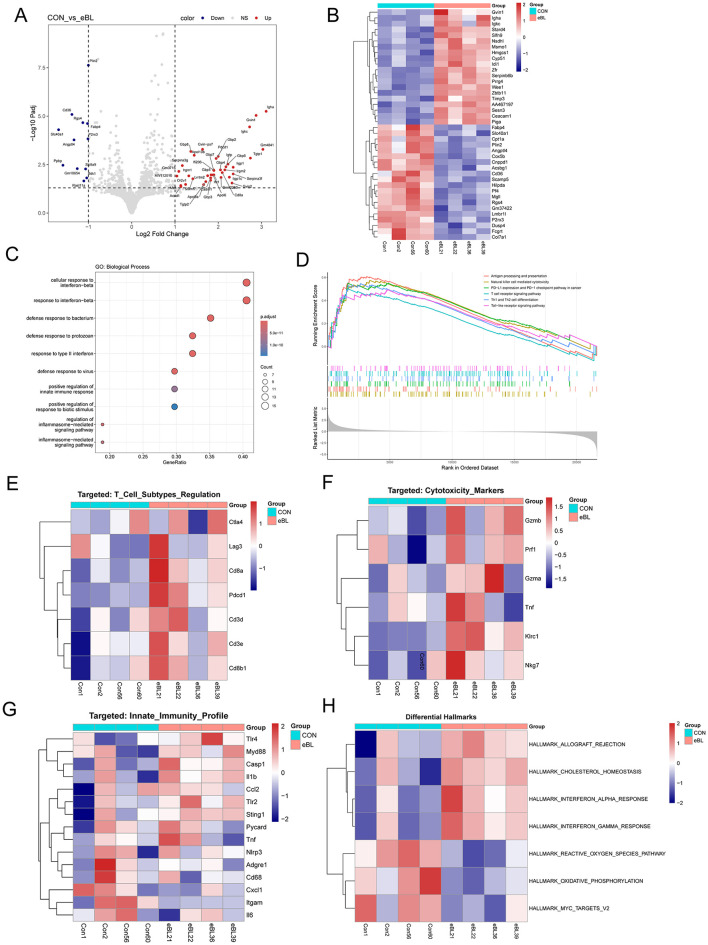
Transcriptomic profiling reveals immune activation and metabolic reprogramming in *B. licheniformis*-treated tumors. **(A)** Volcano plot of differentially expressed genes (DEGs) between PBS-treated (CON) and *B. licheniformis*-treated (eBL) groups (*n* = 4 per group). Red dots represent significantly upregulated genes (Log2FC > 1, p-adj < 0.05) in eBL groups, including cytotoxic markers (*Gvin1, Igha*); blue dots represent downregulated genes (Log2FC < −1, p-adj < 0.05), including metabolic regulators (*Cd36, Fabp4*). **(B)** Heatmap of the top DEGs between CON and eBL groups. The color scale represents Z-scored expression values (Red = High, Blue = Low). **(C)** Gene Ontology (GO) enrichment analysis of upregulated DEGs in eBL group, highlighting key biological processes (BP). **(D)** Gene Set Enrichment Analysis (GSEA) reveals significant activation of immune-related signaling pathways in *B. licheniformis* group. **(E)** Characterization of T-cell infiltration and activation states. Heatmap visualization of pan-T cell (*Cd3d, Cd3e*) and CD8 lineage markers. **(F)** The heatmap displays the normalized expression of genes governing cell-mediated lysis. Note the synchronized upregulation of *Gzmb, Prf1* (Perforin), and *Nkg7* in the *B. licheniformis*-treated group, confirming the presence of functionally active cytotoxic cells within the tumor microenvironment. **(G)** Heatmap of innate immune landscape between CON and eBL groups. **(H)** Heatmap for GSVA enrichment scores for Hallmark gene sets between CON and eBL groups. Each column represents a biological replicate.

To decipher the molecular mechanisms driving tumor suppression, we performed RNA-seq on bulk tumor tissues harvested from the subcutaneous mouse models. Differential expression analysis identified a distinct transcriptional shift in the *B. licheniformis*-treated group as compared to PBS-treated group (Figure 5A). As visualized in the volcano plot and heatmap, the treatment induced the robust upregulation of genes associated with cytotoxic lymphocyte activation, including CD8 antigen alpha (*Cd8a*) and Programmed cell death 1 (*Pdcd1*), corroborating our flow cytometry findings on increased CD8+ T-cell activation and infiltration (Figures 5A, B).

Functional enrichment analysis provided mechanistic insight into how this immune response is triggered. We observed a robust enrichment of “defense response to bacterium” and “positive regulation of innate immune response,” confirming that the tumor microenvironment actively senses the probiotic (Figure 5C). Gene set enrichment analysis (GSEA) further revealed activation of multiple key immune cell pathways, including those involved in both regulation of innate and adaptive immune responses, defense against virus and bacteria, and activation of multiple immune cell pathways, including T cell receptor signaling, cytokine-cytokine receptor interactions, and natural killer (NK) cell-mediated cytotoxicity (Figure 5D).

Further identification of T subtype cell markers showed upregulation of CD8 lineage markers (*Cd8a, Cd8b1*) and pan-T cell markers (*Cd3d, Cd3e*) (Figure 5E). Activation of cytotoxic markers including Gzmb, Prf1, Klrc1 and Nkg7 indicates activation of CD8+ T cells and NK cells (Figure 5F), corroborating findings in flow cytometry and pathway enrichment analysis. Consistent with the “defense response” signature, we observed the upregulation of key innate sensors, including Toll-like receptors (*Tlr*) and the STING pathway components (*Sting1, Cgas*) (Figure 5G). Crucially, this innate priming precipitated a functional immune influx. Lastly, analysis of hallmark immune signatures revealed a distinct activation profile in the treated group, characterized by the enrichment of “interferon alpha/gamma response,” “allograft rejection,” and “cholesterol homeostasis” pathways (Figure 5H). Altogether, integrating these immunological findings with the observed lipid downregulation suggests that *B. licheniformis* remodels the tumor microenvironment via enhancing cytotoxic immune surveillance while concurrently inducing a state of metabolic starvation.

## 4 Discussion

In this study, we identified an elephant gut-derived probiotic, *Bacillus licheniformis*, that suppresses CRC progression by T cell-mediated antitumor immunity and inducing cancer cell cytotoxicity. To our knowledge, this is the first report functionally linking elephant gut microbiota to cancer resistance. The consistent alleviation in gut inflammation and tumor burden across multiple mouse models, combined with its direct anti-proliferative effect on cancer cells, suggest that *B. licheniformis* contributes to the cancer-resistant phenotype of elephants via a mechanism of “functional mimicry,” offering a novel bio-inspired therapeutic avenue for CRC.

Beyond the well-documented cancer-resistant features of elephant genomes (e.g., TP53 expansion), our study unveils a novel dimension of their anti-cancer arsenal rooted in their gut microbiota. Elephants, as herbivores consuming a high-fiber diet, host a specialized gut microbiota optimized for digesting complex plant materials. Our metagenomic analysis revealed a rich consortium of cellulose-degrading taxa, including *Bacillus, Prevotella*, and *Ruminococcus*. These bacteria are essential for breaking down cellulose and other plant polysaccharides into bioavailable nutrients such as SCFAs (Portincasa et al., 2022; Nishiwaki et al., 2022; Liao et al., 2024). Notably, *B. licheniformis* emerged as a persistent gut symbiont in elephants inversely correlated with host age, suggesting its potential role in maintaining gut homeostasis. Genomic characterization of the isolate further revealed enrichment for protease, lipase, and amylase, essential for breaking down polysaccharides into bioavailable nutrients such as SCFAs. We propose that this co-evolved microbial community not only supports host metabolism but also functionally complements the host's genetic adaptations by maintaining a high state of immune surveillance.

Emerging evidence suggests gut microbiota as a pivotal regulator of host immunity (Zheng et al., 2020). Consistent with our findings that DSS disrupts gut barrier integrity and Th17/Treg cell balance and oral administration of *B. licheniformis* restores these damages and cell balance in acute colitis mouse models, recent evidence revealed that gut microbiota regulates the balance of Th17/Treg cells both inside and outside of the intestines and gut dysbiosis can induce systemic immune dysregulation (Wang et al., 2024). Furthermore, in CRC patients, dysbiosis is frequently observed with characteristic increases in pathogenic or pro-inflammatory organisms such as *Fusobacterium nucleatum* and *Porphyromonas* and decreases in probiotic organisms such as *Bifidobacterium* and *Lactobacillus* (Giri et al., 2022; Gu et al., 2020; Wong and Yu, 2019; Ren et al., 2021).

Bacterial species influence CRC progression through direct and indirect mechanisms (Li et al., 2021; Lei et al., 2025). Our study showed that elephant gut microbiota-derived *B. licheniformis* exhibited potent anti-inflammatory and immunomodulatory properties in CRC treatments by activating tumor-infiltrating immune cells such as cytotoxic CD8+ T cells. The soluble metabolites produced by this strain can directly induce cytotoxicity and upregulate the tumor suppressor p53 in human colorectal cancer cells, suggesting a crucial potential mechanism to complement its immune-mediated effects. Many probiotic strains, including *Bifidobacterium* and *Lactobacillus*, have been shown to improve immunity and cancer suppression by modulating the gut microbiota and enhancing various immune responses (Pei et al., 2024). Our previous study showed that *Lactobacillus plantarum* and its metabolite indole-3-lactic acids significantly suppressed CRC progression by activating tumor-infiltrating CD8^+^ T cells (Zhang et al., 2023). The discovery that *B. licheniformis* possesses both immunomodulatory and direct cytotoxic effects positions it as a uniquely promising therapeutic candidate. Many of these beneficial organisms are short-chain fatty acid producers that regulate gut homeostasis and maintain the integrity of the mucosal barrier. Consistent with these results, *B. licheniformis* is also known as a SCFA producer that remodels gut microbiota and mitigates systemic inflammation, suggesting its potential beneficial role in regulating CRC and immune responses. Notably, our genomic analysis suggests elephant-derived *B. licheniformis* possesses enhanced capabilities for valeric acid production, which exhibits therapeutic effects in colonic diseases.

Consistent with results in mouse models, RNA-seq analysis of tumor tissues in this study revealed that *B. licheniformis* administration remodels the immune landscape of colorectal cancer. In particular, immune-related genes and pathways were activated, including those associated with tumor-infiltrating cytotoxic T cells. These results highlighted the potential roles of these immune cells in activating anti-tumor effects. CD8+ T cells are crucial in the adaptive immune response to cancer, and their infiltration in tumor predicts positive outcomes (Zhang and Zhang, 2020). This is consistent with our mouse models that revealed an increase in tumor-infiltrating GZMB+ CD8+ T cells. These cells represent a highly activated cytotoxic group with high capability to target and eradicate tumor cells (Santos-Zas et al., 2021; Bassez et al., 2021; Zhang et al., 2024). In one recent study, oral administration of a traditional Chinese medicine (Huaier) showed changes in gut microbiota composition along with an increase in GzmB^+^CD8^+^ T cells in the tumor microenvironments (Wang et al., 2025).

While this study provides compelling evidence for the therapeutic potential of elephant-derived *B. licheniformis* regarding CRC treatment, investigating the precise underlying mechanisms by which *B. licheniformis* exerts its anti-inflammatory and anti-cancer effects is crucial. This involves further elucidating how specific probiotic-derived metabolites modulate p53 upregulation and cytotoxicity, and the signaling pathways that are modulated by the probiotic, especially their specific effects on the infiltration and function of CD8+ T cells and the direct suppression of cancer cell proliferation. The unique biodiversity of animal microbiome, particularly in species with exceptional health traits like elephants, offers a largely untapped resource for exploring novel therapeutic agents satisfying medical needs in a range of diseases (Levin et al., 2021).

## Data Availability

The raw sequencing data generated in this study, including whole genome sequencing, metagenomics, and RNA-seq datasets, have been deposited in the National Center for Biotechnology Information (NCBI) BioProject database under accession number (PRJNA1415237).
