# Supplementary figures and images for: Elephant-derived Bacillus licheniformis modulates immune cells shedding light on cancer resistance
Source: Front Microbiol. 2026 Apr 1;17:1753410. doi: 10.3389/fmicb.2026.1753410 (PMC13079344; doi:10.3389/fmicb.2026.1753410)

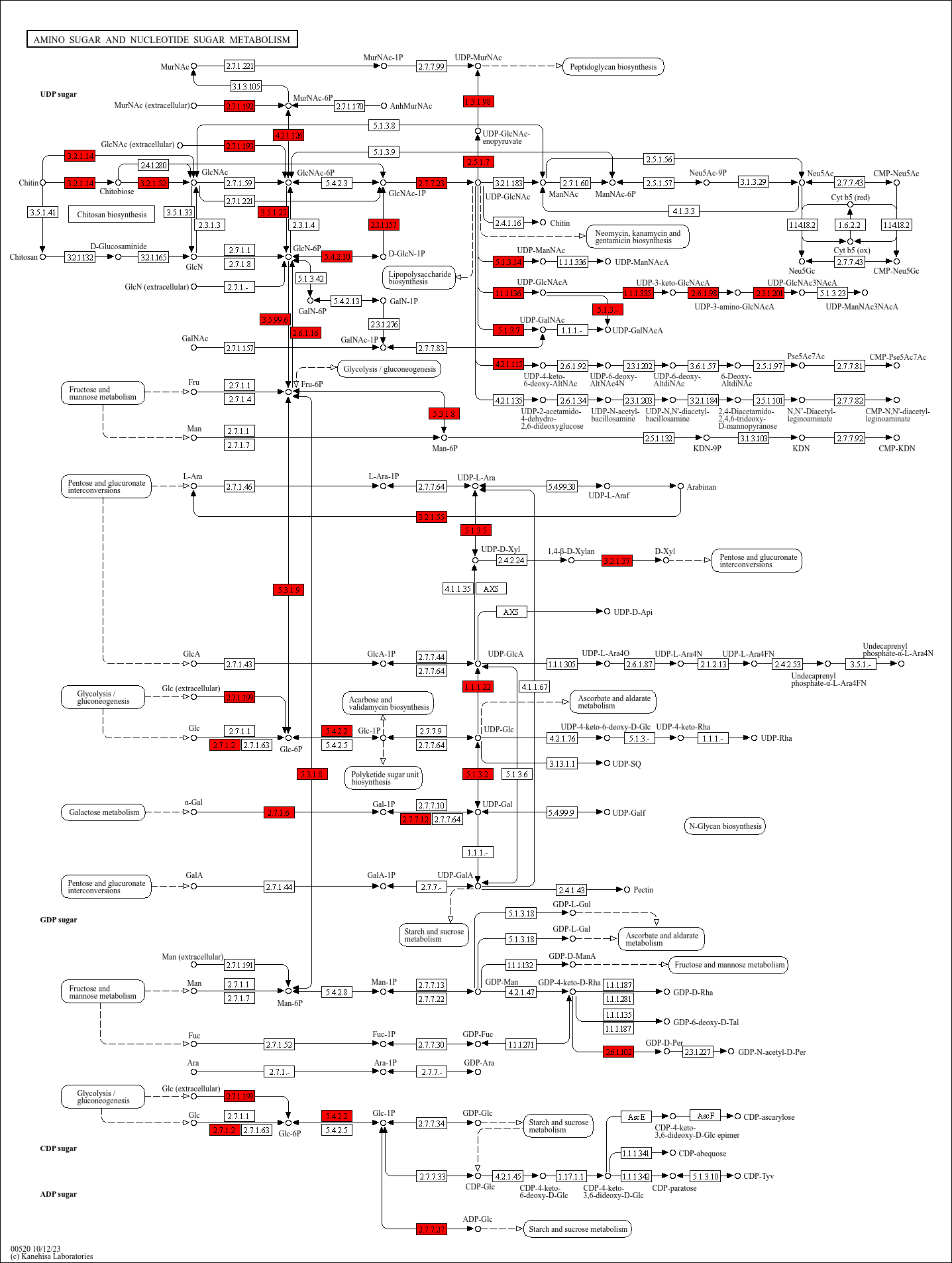

Supplement: Supplementary file 2 [file Image_1.tif]

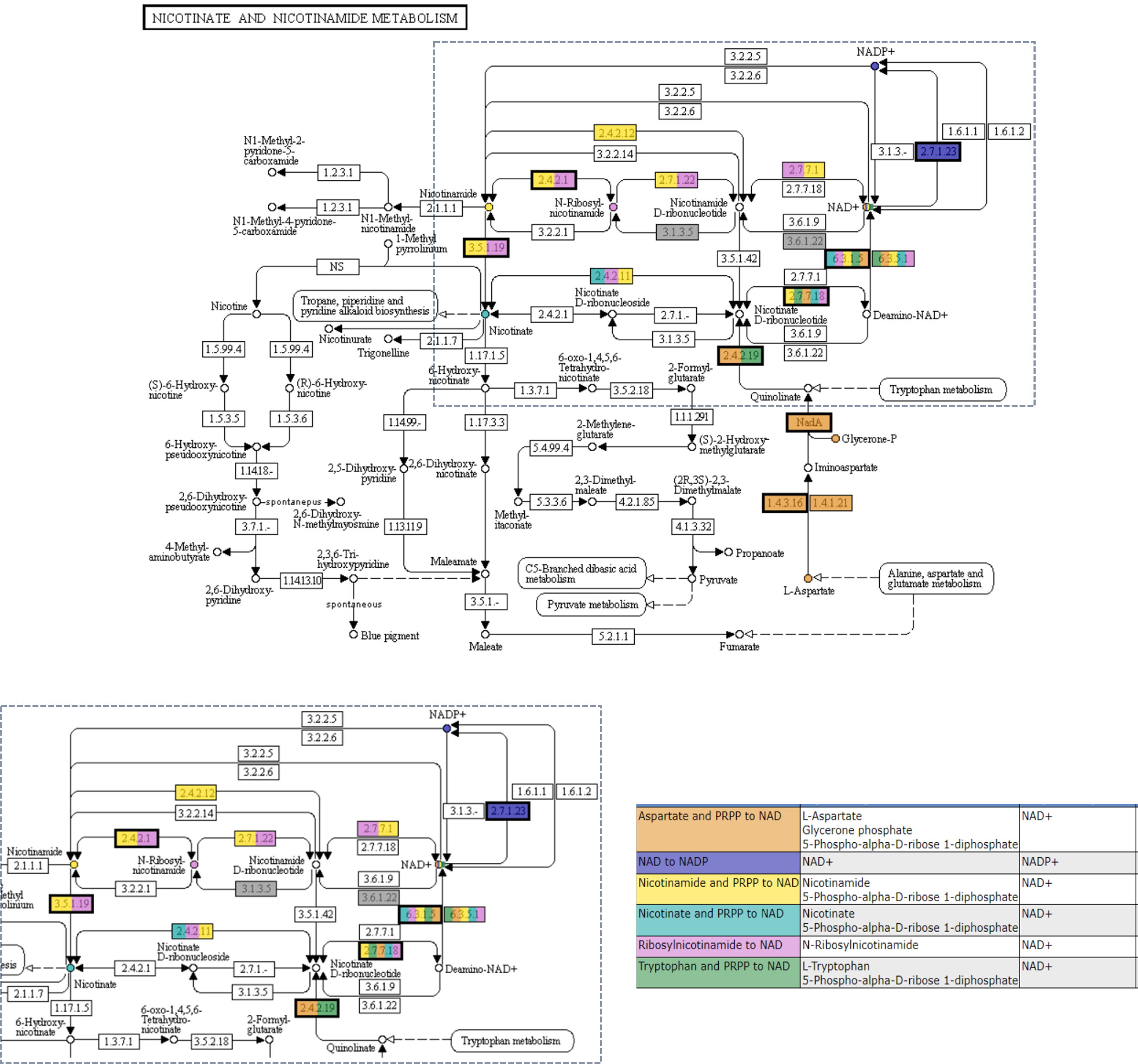

Supplement: Supplementary file 3 [file Image_2.tif]
